# Shape and size of the arenas affect amphipod behaviours: implications for ecotoxicology

**DOI:** 10.7717/peerj.5271

**Published:** 2018-07-26

**Authors:** Shanelle A. Kohler, Matthew O. Parker, Alex T. Ford

**Affiliations:** 1 Institute of Marine Sciences, Biological Sciences, University of Portsmouth, Portsmouth, United Kingdom; 2 School of Pharmacy and Biomedical Science, University of Portsmouth, Portsmouth, United Kingdom

**Keywords:** Behaviour, Amphipod, Ecotoxicology, Crustacea, Thigmotaxis, Phototaxis, Anxiety behaviours

## Abstract

The use of behaviour in ecotoxicology is expanding, however the lack of standardisation and validation of these assays currently presents a major drawback in moving forward in the development of behavioural assays. Furthermore, there is a current paucity of control data on test species, particularly invertebrate models. In this study we assessed a range of behaviours associated with spatial distribution and locomotion in relation to arena size and shape in two species of amphipod crustacean (*Echinogammarus marinus* and *Gammarus pulex*). Arena shape had significant effects on almost all behavioural parameters analysed. Increasing arena size resulted in an increased mean velocity and activity plus increased proportional use of the central zones. These results indicate that ‘ceiling effects’ may occur in some ecotoxicological studies resulting in potentially ‘false’ negative effects if careful consideration is not paid to experimental design. Differences in behaviours were observed between the two species of amphipod. For example, *G. pulex* spend approximately five times (∼20%) more of the available time crossing the central zones of the arenas compared to *E. marinus* (∼4%) which could have implications on assessing anxiolytic behaviours. The results of this study highlight several behaviours with potential for use in behavioural ecotoxicology with crustaceans but also underscore the need for careful consideration when designing these behavioural assays.

## Introduction

Behaviour is a useful endpoint in ecotoxicology as it may provide a link between biochemical and ecological effects of environmental contamination (Scott & Sloman, 2004; Sloman & Mcneil, 2012). In recent years, the use of behavioural endpoints in toxicity studies has increased, facilitated by advancements in computational equipment and automaton, allowing for the sensitive analysis of a variety of complex behavioural end points such as those associated with stress and anxiety (Klaminder et al., 2016; Spink et al., 2001; Pittman & Ichikawa, 2013; Cachat et al., 2011). “Anxiety-like” behaviours are diverse and include scototaxis such as preference for dark environments (Maximino et al., 2010a; Maximino et al., 2010b; Maximino et al., 2011), sheltering and exploratory behaviours (Perrot-Minnot, Banchetry & Cézilly, 2017; Baiamonte et al., 2016), thigmotaxis ‘wall-hugging’ (Schnörr et al., 2012; Baiamonte et al., 2016), changes in activity and mobility (Martin et al., 2017; Cachat et al., 2010), behaviour towards conspecifics such as aggression (Bacqué-Cazenave et al., 2017; Perreault, Semsar & Godwin, 2003) and social cohesion (Parker et al., 2014; Miller & Gerlai, 2007). Many of these endpoints have been assessed by pharmacological studies and have previously been limited to vertebrate models namely rodents and fish. However, recent advances have shown that anxiety-like behaviours are also present in some invertebrate species (Perrot-Minnot, Banchetry & Cézilly, 2017; Fossat et al., 2014; Bacqué-Cazenave et al., 2017; Tierney et al., 2016). Anxiety-like behaviours have been used for the assessment of environmental contaminants, including pharmaceuticals that act to modulate behaviour (Hamilton et al., 2016; Wong, Oxendine & Godwin, 2013; Egan et al., 2009). However, studies have been met with some scepticism as to their repeatability due to varied results found in the literature (Sumpter, Donnachie & Johnson, 2014). This is potentially due to a lack of standardisation and validation of behavioural assays for the assessment of behavioural endpoints (Spruijt et al., 2014; Parker, 2016; Melvin et al., 2017). For example, the open field test has historically been implemented to measure multiple behaviours associated with anxiety and to assess the effects of behavioural modifying compounds in rodents. However, despite its ubiquitous use in behavioural studies the test has been reported as having low validity due to a lack of standardisation (Spruijt et al., 2014; Haller & Alicki, 2012). Factors including both the shape and size of the arena, light conditions and definition of zones have been identified as things to consider when conducting these tests (Grabovskaya & Salyha, 2014; Eilam, 2003; Spruijt et al., 2014). In addition to a lack of standardisation, the use of behavioural endpoints in ecotoxicology and their translation to other species is currently limited by our understanding of the baseline behaviours of many model organisms, and the relevance of these behaviours to higher level effects (Melvin & Wilson, 2013; Parker, 2016). Thigmotaxis or ‘wall hugging’ is a conserved behaviour whereby organisms placed in a novel environment will stay close to walls or corners and has been demonstrated in mice (Simon, Dupuis & Costentin, 1994; Treit & Fundytus, 1988), zebrafish (Richendrfer et al., 2012; Schnörr et al., 2012; Baiamonte et al., 2016) and crayfish (Tierney et al., 2016) as a viable endpoint for behavioural study. Amphipods have been used extensively in ecotoxicological studies for a range of endpoints associated with lethality, feeding, reproduction, behaviour and for biomarker studies (see Kunz, Kienle & Gerhardt, 2010 and references within). They are ubiquitous in almost all aquatic systems, covering a wide trophic range as herbivores, detritovores and predators, as well as constituting an important prey for many fish species (Glazier, 2009). Some amphipod species have been used successfully in ecotoxicology assays for the assessment of phototactic behaviour (Guler & Ford, 2010), activity (Bossus et al., 2014) and anxiety (Perrot-Minnot, Banchetry & Cézilly, 2017). These are conserved behaviours which act to maintain fitness in an organism and a reduction in anxiety behaviours have been demonstrated to influence predation risk (Perrot-Minnot, Banchetry & Cézilly, 2017; Dianne et al., 2011; Bauer et al., 2005; Bethel & Holmes, 1973). To date, the thigmotactic behaviours in amphipods are currently unknown, and it is unclear or how the occurrence of thigmotaxis relates to anxiety. Recently, Perrot-Minnot, Banchetry & Cézilly (2017) found that amphipods when exposed to electric shocks would demonstrate increased refuge behaviours. These refuge behaviours were mitigated by the exposure to anxiolytic drugs suggesting that they might exhibit anxiety based behaviours.

When designing new behavioural assays for aquatic toxicology there is ultimately a trade-off between high-throughput analysis and the size of experimental arenas. For example, there is a risk posed by creating arenas that are too small for the model organism in that the behaviours one looks to measure could be constrained, restricting the organisms ability to behave ‘normally’. Conversely, creating unnecessarily large experimental arenas requires greater lab resources (e.g., space) and can ultimately impact the speed at which organisms can be monitored which may impact replication. The shape of the arenas might also have impacts on spatial distribution, time spent active (i.e., use of refuge areas vs exploratory behaviours) or patterns of swimming.

In this study, a method for measuring swimming speed, activity and thigmotaxis behaviours are presented using two amphipod species, *Echinogammarus marinus* and *Gammarus pulex*, representing a marine and freshwater model. *E. marinus* is an intertial, esturine species of amphipod comprising an important food source for wading birds (Múrias et al., 1996; Martins, Maranhão & Marques, 2002). *G. pulex* represents a freshwater species ubiquitous in most freshwater systems and providing an important role as a detritovore and prey species (Welton, 1979; Maitland, 1966). The main aims of the study were to determine whether the shape and size of an arena have an effect on thigmotaxis, spatial usage within arenas, swimming speeds and activity. In this way, we hope to provide information on some of the baseline behaviours of two amphipod species for use in behavioural ecotoxicology and to contribute towards the standardisation of behavioural assays.

## Methodology

### Specimen collection and husbandry

*E. marinus* were collected from Langstone Harbour, Portsmouth, UK (co-ordinates 50°47′22.8″N, 1°02′35.9″W) at low tide and transported back to the Institute of Marine Sciences, Portsmouth, UK. Specimens were acclimated for one week in filtered seawater from Langstone Harbour at 10 ± 1 °C under a 24-hour dark photoperiod as this is sufficient to remove alterations in activity due to circadian rhythms in this species (Kohler et al. unpublished data). *Fucus vesiculosis* or *Ascophyllum nodosum* was provided as both a food source and substrate. *G. pulex* were collected from the river Ems, Emsworth, UK (co-ordinates 50°51′40.5″N, 0°55′42.5″W). Specimens were caught in a 1 mm mesh net using the kick sampling method outlined by the freshwater biological association (FBA, 2015). Organisms were transported back to the Institute of Marine Sciences, Portsmouth, UK and left to acclimate to laboratory conditions for one week in Ems river water at 10 ± 1 °C under a 24-hour dark photoperiod for consistency with *E. marinus* experiments. Leaf litter collected from the sample site acted as a natural substrate and food source. Prior to acclimation, adult males of both *E. marinus* and *G. pulex* were selected for behavioural studies. Organisms were individually assessed under a backlight and only healthy specimens were selected. Juveniles and specimens suffering the loss of limbs or with visible parasites were excluded from the study.

### Analysis of behaviour

Following the one-week acclimation period, the behaviours of both *E. marinus* and *G. pulex* were analysed using a DanioVision™ observation chamber connected to EthoVision^®^XT 11.5 video tracking software (TrackSys, Nottingham, UK). The observation chamber supports an infrared camera mounted above an internal holder for an arena plate or small container. The internal holder is backlit by both infrared and an additional white light. Prior to behavioural assays, individuals were gently transferred from holding tanks to arena plates containing tank water and allowed to acclimate to test conditions for one minute in the DanioVision™ observation chamber. A plastic spoon was used for the transfer of organisms in tank water to minimise handling stress which may impact behaviours (Gouveia & Hurst, 2017; Thompson et al., 2016). Organisms were then tracked for a total of 8 min under a 2-minute dark—2-minute light cycle. ‘Light On’ was used as a disturbance to induce a behavioural response, and replicates were done under a range of light intensities, 100%, 50%, 20% & 5% (4000, 2000, 800 & 200 lux respectively).

### Experimental design rationale

**Figure 1:**
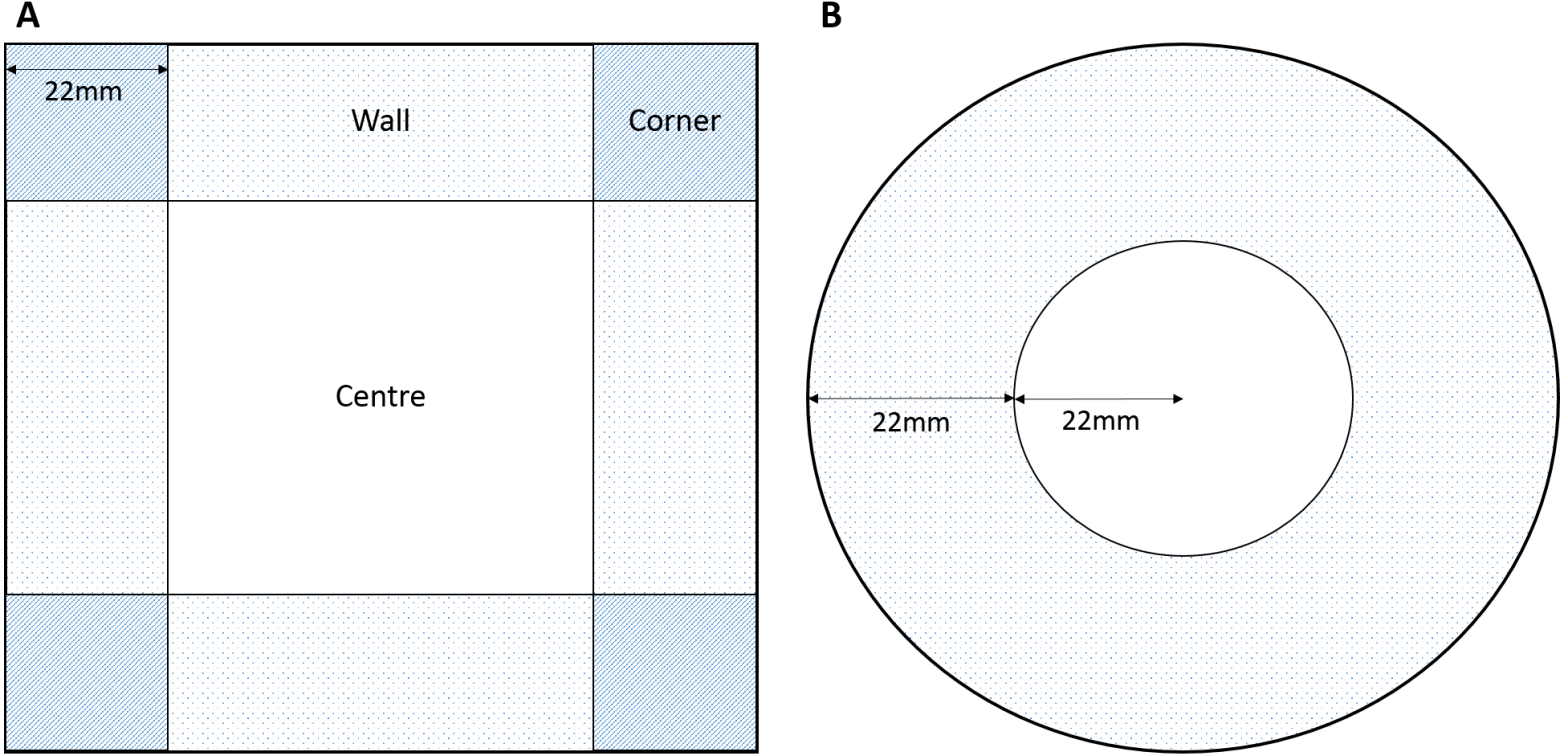
Dimensions of zones for (A) square and (B) round arenas.

The rationale behind the choice of arena sizes focussed in the need to fit within the Daniovision™ hardware therefore we focused on sizes approximate to a standard multi-well plate or petri dish. Due to the propensity for amphipods to swim around the outside of a curved (round) petri dish we also introduced a square plate to interrupt the continuous swimming pattern, create a ‘refuge’ and potentially evoke increased centre use. A square 100 × 100 mm petri dish (Fig. 1A) and a standard round petri dish (Fig. 1B) were used to assess the effects of round or square arenas on behaviour. Central and outer zones were marked in the EthoVision^®^XT software (Fig. 1). The width of the external zone was calculated from the round petri dish as a quarter of the circumference. This allowed for the outer zone to be the same width as the radius of the central zone. The width of the external zones of the square petri dish were kept the same as that of the round dish for consistency in outer zones.

**Figure 2:**
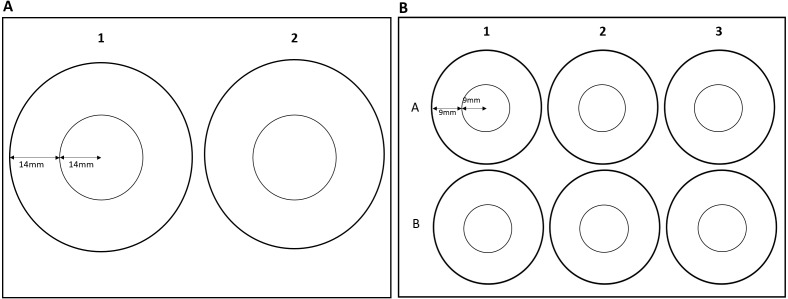
Dimensions of zones for (A) two-well plate and (B) six-well plate.

The effects of zone use on thigmotaxis and swimming parameters were also assessed for *G. pulex* using round arenas only. Effects of arena size could not be assessed for *E. marinus* due to their larger size, resulting in individuals occupying both the inner and outer zones at the same time in smaller arenas. *G. pulex* were tracked as described above in a two-well and a six-well plate (Fig. 2). Central and outer zones were calculated as described above so that the area of the central and outer zones would remain in the same proportion for each size class (Table 1). Zones were marked out with the EthoVision^®^XT software (Figs. 2A and 2B).

**Table 1:** Dimensions of arenas used through the duration of the study for all arena sizes used. In Total dimensions (mm), r represents radius of round arenas. Percent data represents the percentage of the centre zone as a proportion of the total arena area.

	Total dimensions (mm)	Area (mm^2^)		Percent area (centre zone)
		Inner zone	Outer zone	
Square	100 × 100	3,136.0	6,864.0	45.0%
Round (1-Well)	44r	1,520.5	4,561.6	33.3%
Round (2-Well)	28r	615.6	1,847.3	33.3%
Round (3-Well)	18r	254.5	763.4	33.3%

### Subjects

A total of 768 animals were analysed in the tracking system. For *E. marinus* 120 replicates were performed per arena shape (i.e., round and square). For *G. pulex* 120 replicates were performed per arena shape and a further 144 replicates per arena size (i.e., two-well and six-well plate). The University of Portsmouth granted ethical approval to carry out the study within its facilities.

### Statistics

Zone use was measured as a comparison of the percent duration of time spent in central and outer zones. Swimming speed was measured as mean velocity per second and activity was calculated as the percentage of time an organism spent mobile compared to immobile. Statistical analysis was performed in IBM SPSS Statistics 24. Linear mixed effects models were used for all comparisons. Total distance was used as a co-variate for the analysis of thigmotactic behaviour to correct for animals that did not move during trials. Extreme anomalous values generated by the loss of tracking by the EthoVision^®^XT software were removed from the data analysis and never represented more than 3% of the total datasets. Bonferroni corrections were carried out to characterise main effects and interactions. *P*-values of <0.05 were considered significant. No differences were observed between light intensity for all endpoints so data were combined for presented figures.

## Results

### Echinogammarus marnius

**Figure 3:**
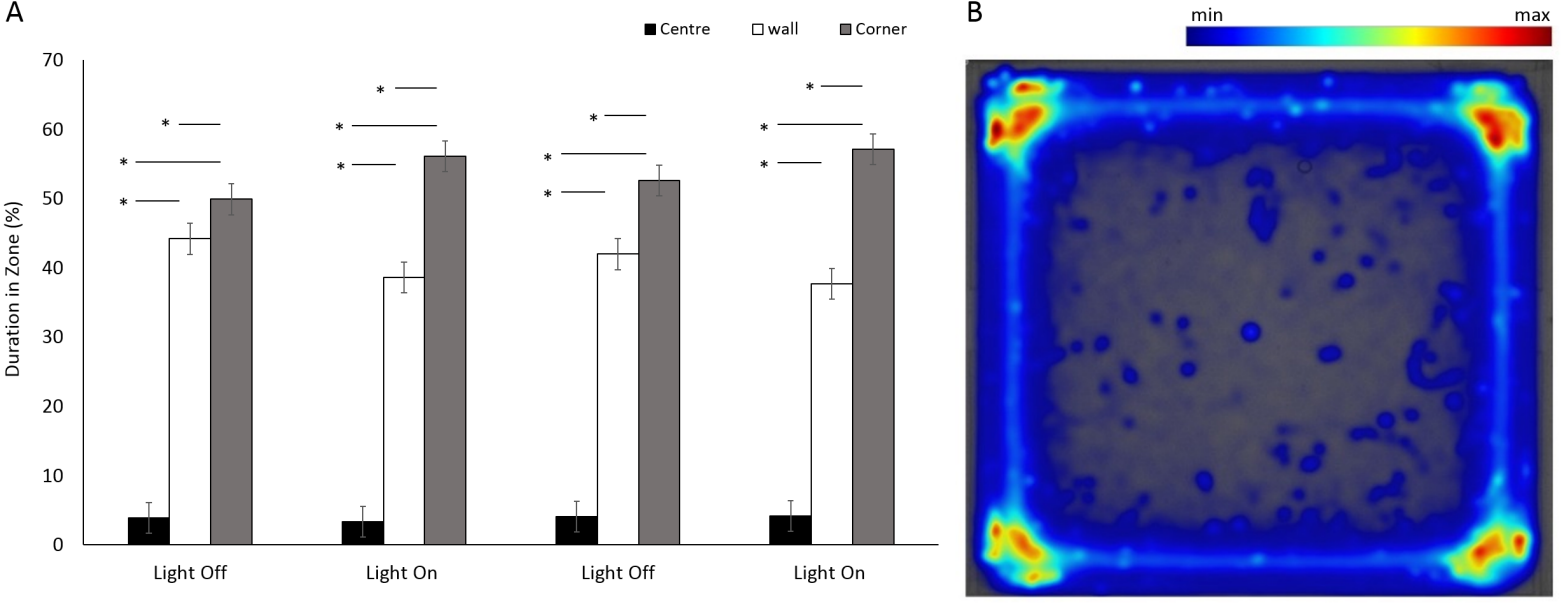
Percent duration of time *E. marinus* spent in central and outer zones between light and dark phases, and heatmap of mean zone use for *E. marinus* in a square arena. (A) Percent duration of time *E. marinus* spent in central and outer zones between light and dark phases. Asterisks represent significant differences between zones (*p* < 0.05). Error bars represent 95% confidence interval. (B) Heatmap of mean zone use for *E. marinus* in a square arena. Data was calculated in EthoVision^®^ XT as mean duration of time spent in an area for all trials.

There was a significant difference between the use of zones (*F*(2, 349.5) = 952.75, *p* < 0.001) in a square arena (Figs. 3A and 3B), with animals spending 95% of the time in a corner or against a wall than in the centre. No significant differences were observed between the four time bins when the light was on or off (*F*(3, 1062.6) = 0.9, *p* = 0.965). However, there was a significant interaction between time bins and zones as a result of increased use of corners when the lights were on (*F*(6, 1036.8) = 13.29, *p* < 0.001; Table S1).

**Figure 4:**
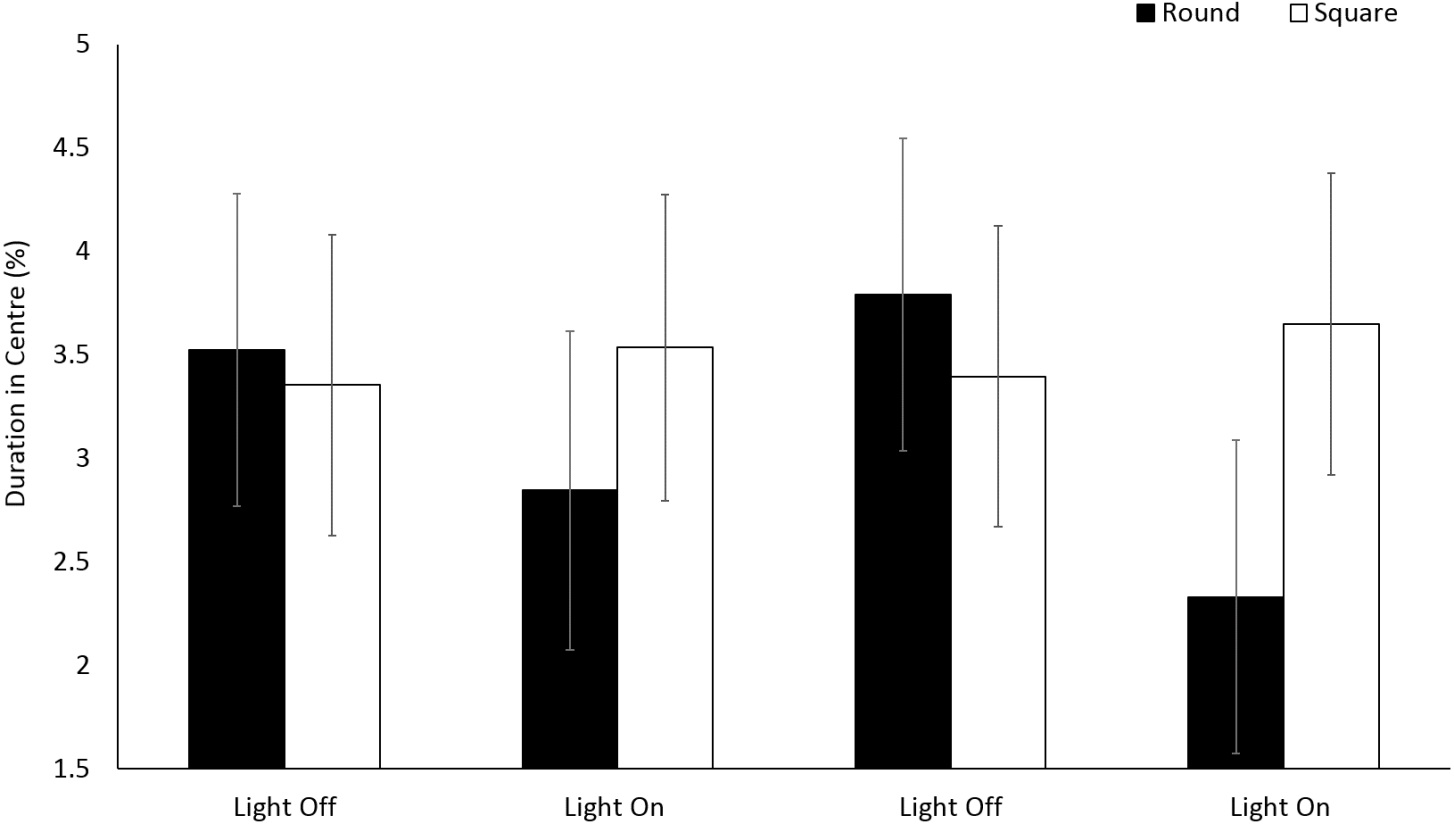
Percent duration of time *E. marinus.* spent in the central zone during light and dark phases. Error bars represent 95% confidence interval.

No significant differences (*F*(1, 196.2) = 1.20, *p* = 0.275) were observed in the use of centre space for *E. marinus* between round and square arenas (Fig. 4). Organisms spent 4% of the time in the central zones for the duration of the trials. No significant differences were observed in the use of central zones over time during light and dark phases (*F*(3, 716.1) = 1.13, *p* = 0.335).

**Figure 5:**
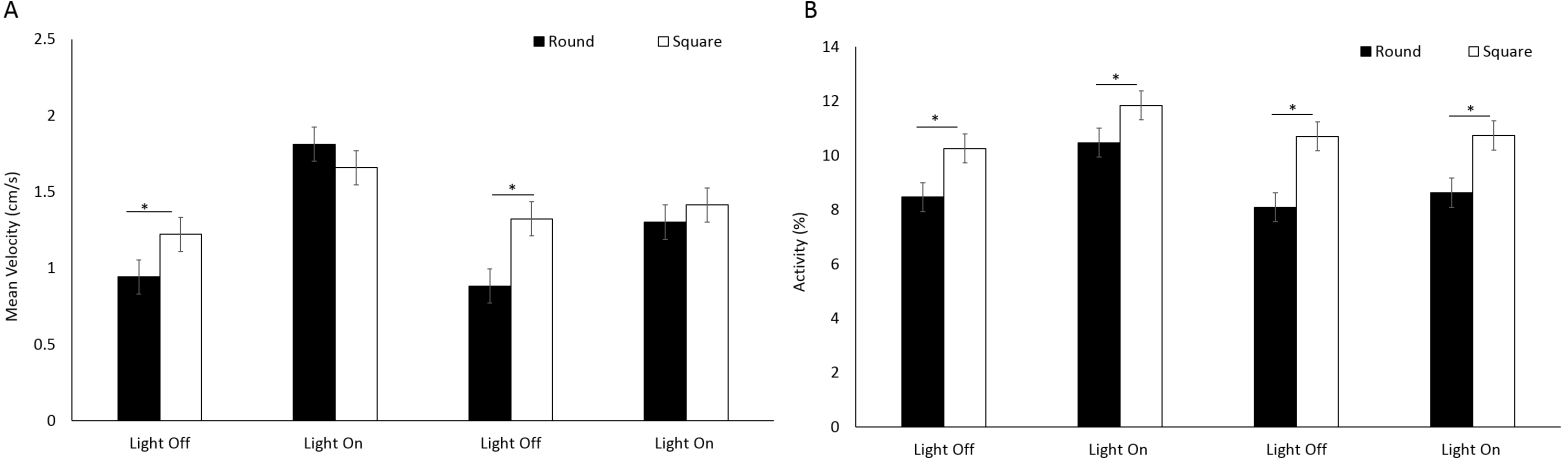
(A) Mean velocity (cm/s) and (B) percent activity of *E. marinus* between round and square arenas during light and dark phases. Asterisks represent significant differences between arena shape (*p* < 0.05). Error bars represent 95% confidence interval.

There was a significant difference in mean velocity between arena shapes (*F*(1, 205.1) = 8.96, *p* = 0.003) (Fig. 5A) and between time bins (*F*(3, 717.1) = 83.76, *P* < 0.001) where velocity was greater during light phases compared to dark (Table S2) A significant interaction between shape and time was observed as a result of greater mean velocity being reached in a square well during light cycles (*F*(3, 717.1) = 14.57, *P* < 0.001; Table S2). Activity was greater overall in square arenas compared to round (*F*(1, 233) = 42.966, *p* < 0.001) irrespective of light or dark cycles (Fig. 5B).

### Gammarus pulex

**Figure 6:**
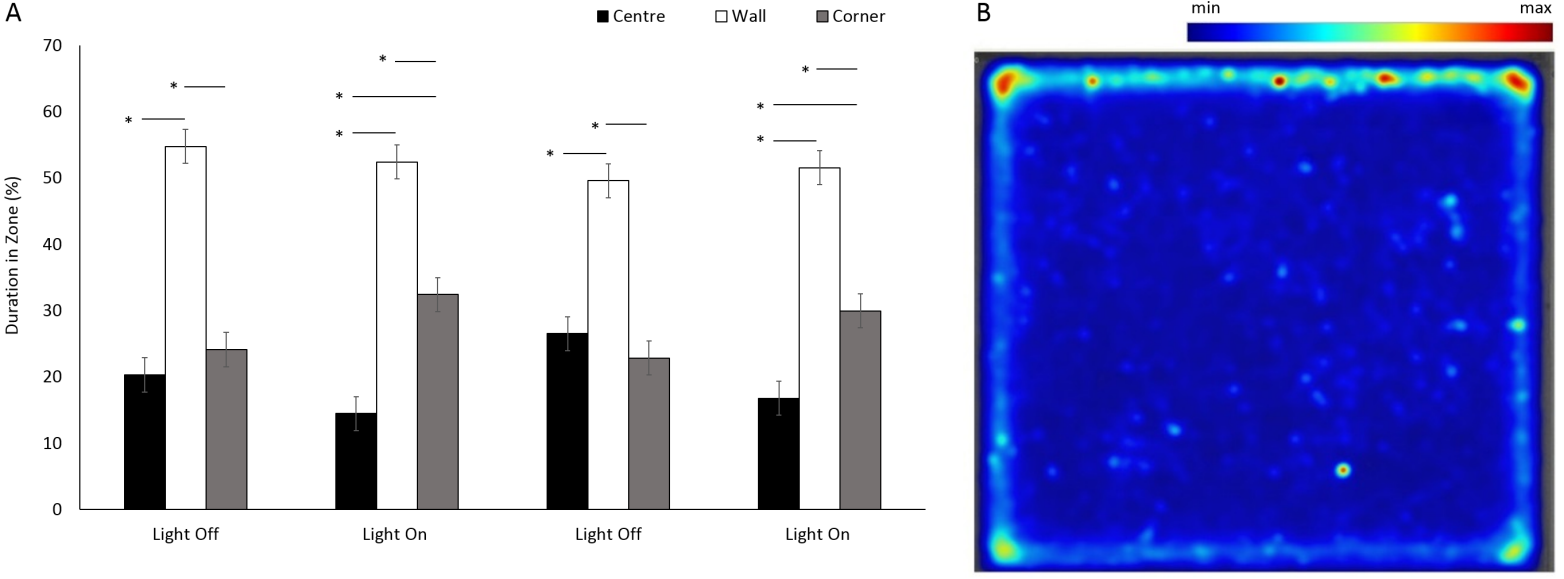
Percent duration of time *G. pulex* spent in central and outer zones during light and dark phases, and heatmap of mean zone use of *G. pulex* for square arena. (A) Percent duration of time *G. pulex* spent in central and outer zones during light and dark phases.** Asterisks represent significant differences between zones (*p* < 0.05). Error bars represent 95% confidence interval. (B) Heatmap of mean zone use of *G. pulex* for square arena. Data was calculated in EthoVision^®^ XT as mean duration of time spent in an area for all trials.

There was a significant difference between the use of zones (*F*(1, 205.1) = 8.96, *p* < 0.001) in a square arena (Figs. 6A and 6B), with animals spending 75% of the time in a corner or against a wall than in the centre. No significant differences were observed between the four time bins when the lights were on and off (*F*(3, 1177.9) = 0.05, *p* = 0.984). However, there was a significant interaction between time bins and zones as a result of decreased use of the centre and an increased use of corners when the lights were on (*F*(6, 1070) = 20.36, *p* < 0.001; Table S1).

**Figure 7:**
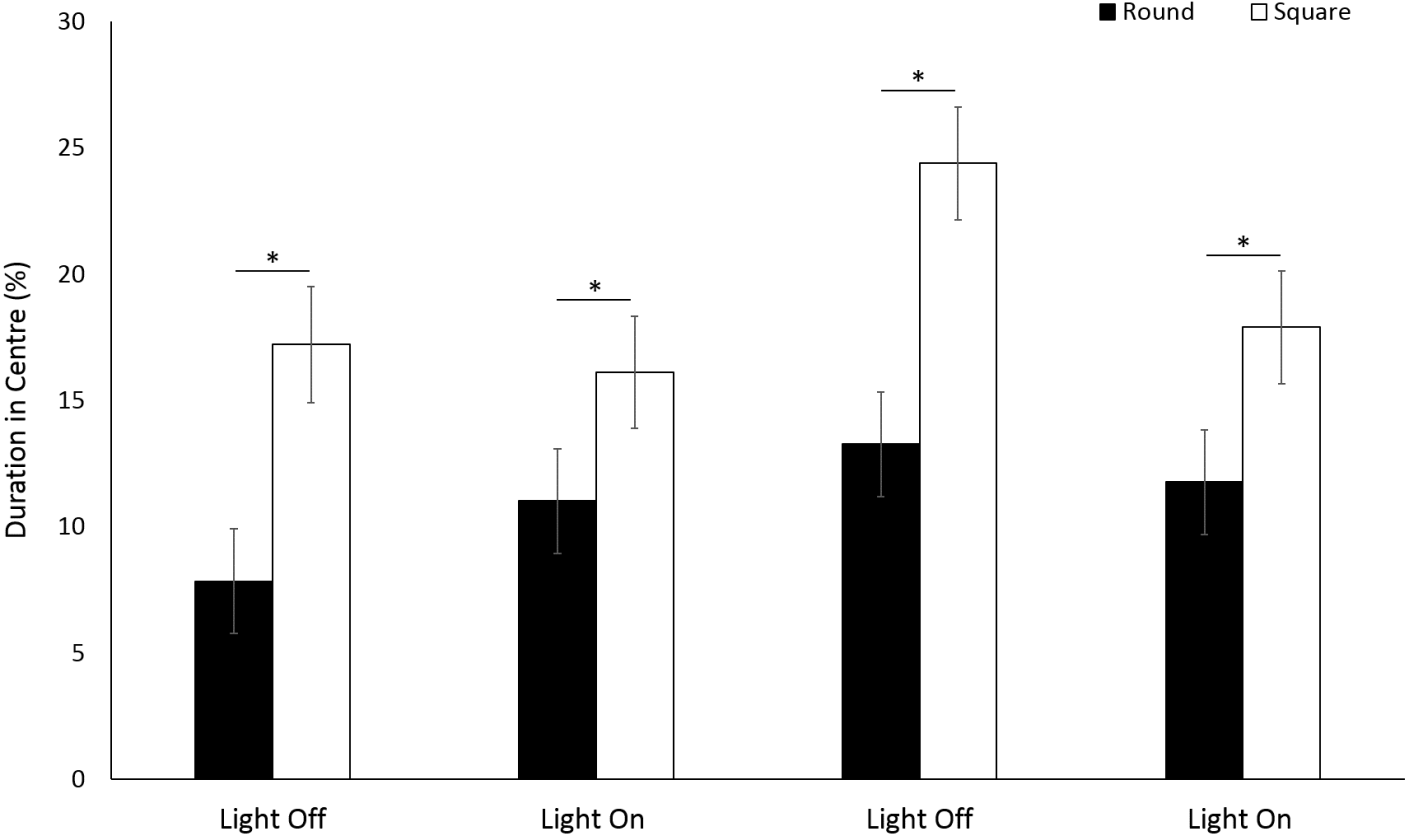
Percent time *G. pulex* spent in the centre zone between light and dark phases. Asterisks represent significant differences between arena shape (*p* < 0.05). Error bars represent 95% confidence interval.

Use of the centre space was significantly greater in a square arena compared to round (*F*(1, 213) = 58.18, *p* ≤ 0.001) for *G. pulex.* (Fig. 7). Significant differences were observed with time between light and dark cycles driven by a greater use of the centre zone during the second dark phase (*F*(3, 835) = 18.27, *p* < 0.001; Table S2). There was a significant interaction between shape and time, whereby the difference in the use of centre space between round and square arenas approximately doubled (±0.9%) during dark phases compared to light (*F*(3, 756.1) = 4.89, *p* = 0.002).

**Figure 8:**
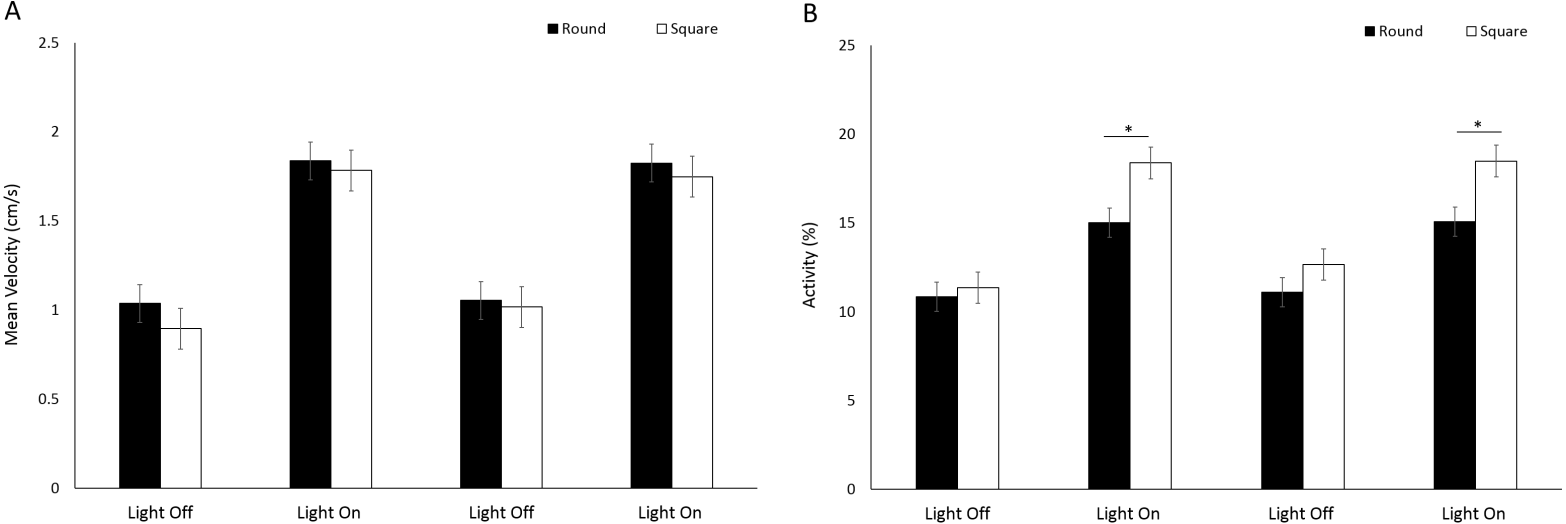
(A) Mean velocity (cm/s) and (B) percent activity of *G. pulex* in round and square arenas during light and dark phases. Asterisks represent significant differences between arena shape (*p* < 0.05). Error bars represent 95% confidence interval.

No significant difference (*F*(1, 224.9) = 1.39, *p* = 0.240) in mean velocity was observed between arena shapes (Fig. 8A). Velocity was significantly greater during light phases compared to dark (*F*(3, 776.1) = 317.036, *p* < 0.001; Table S2). There was a significant difference in activity between arena shapes (*F*(1, 252) = 16.63,  *p* < 0.001), and with time (*F*(3, 756) = 341.22, *p* < 0.001) as a result of increased activity during light phases compared to dark (Fig. 8B). There was a significant interaction between arena shape and time as a result of increased activity in square arenas compared to round during light phases (*F*(3, 756) = 18.49, *p* < 0.001; Table S2).

**Figure 9:**
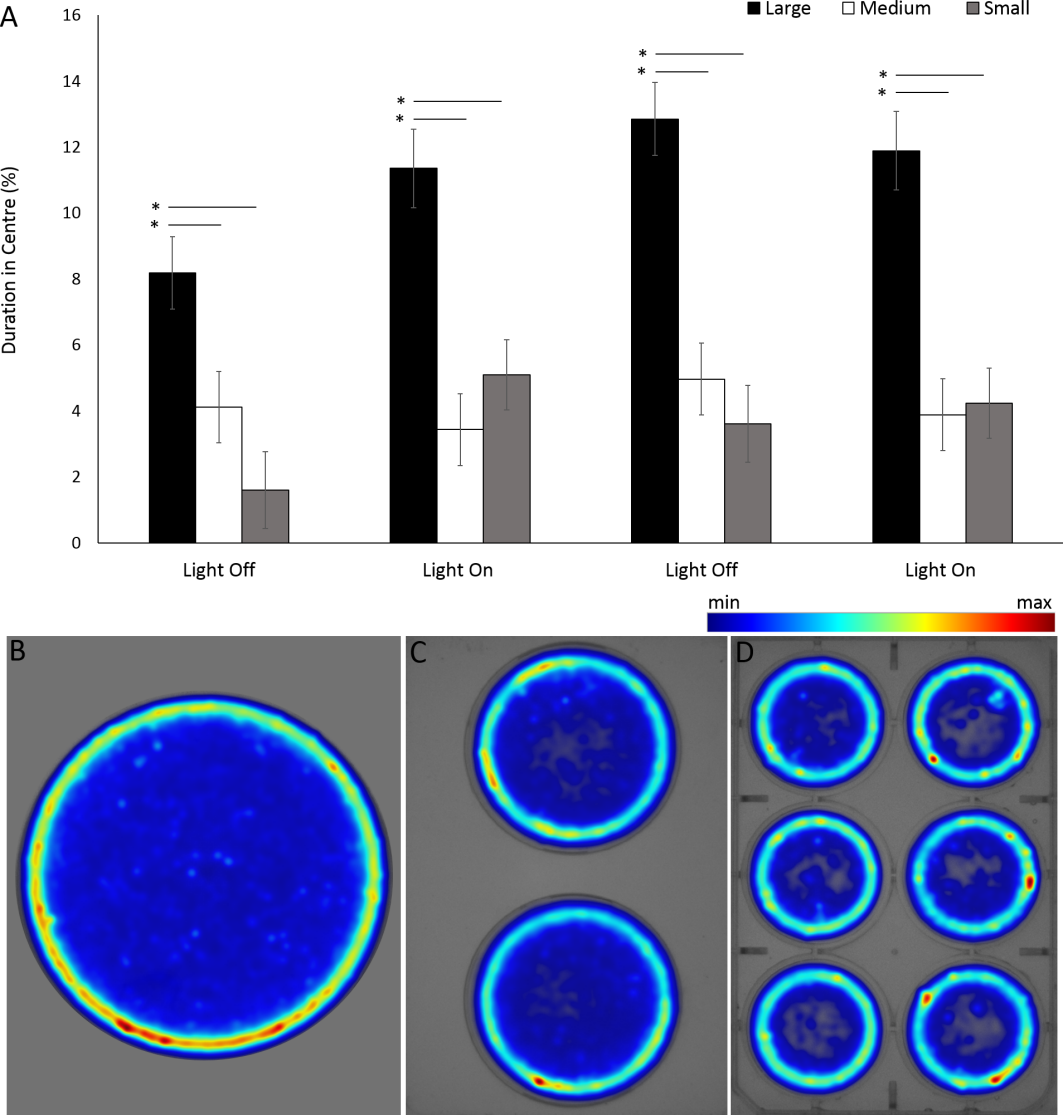
Percentage of time *G. pulex* spent in the central zone between arena sizes during light and dark cycles (A), and heatmaps of zone use for (B) large, (C) medium and (D) small arenas. Asterisks represent significant differences between arena size (*p* < 0.05). Error bars represent 95% confidence interval. Heatmaps were calculated in EthoVision XT as mean duration of time spent in an area for all trials combined.

For the assessment of arena size on behaviours in *G. pulex*, the single petri dish, two-well plate and six-well plate will be henceforth referred to as large, medium and small arena for ease of writing. *G. pulex* spent a significant proportion of the overall time in the centre zone in a large arena compared to medium and small (*F* = 104.321, *df* = 2, *p* < 0.001; Table S3) (Figs. 9A, 9B), despite the area of the zones being proportionally the same between all arena sizes (inner zone 33.3% total area). There was a significant effect of time on duration in the centre (*F*(3, 1302.2) = 16.50, *p* < 0.001) and a significant interaction between arena size and time as a result of increased time spent in the centre zone after the first dark cycle (*F*(6, 1160) = 7.19, *p* < 0.001; Table S3).

**Figure 10:**
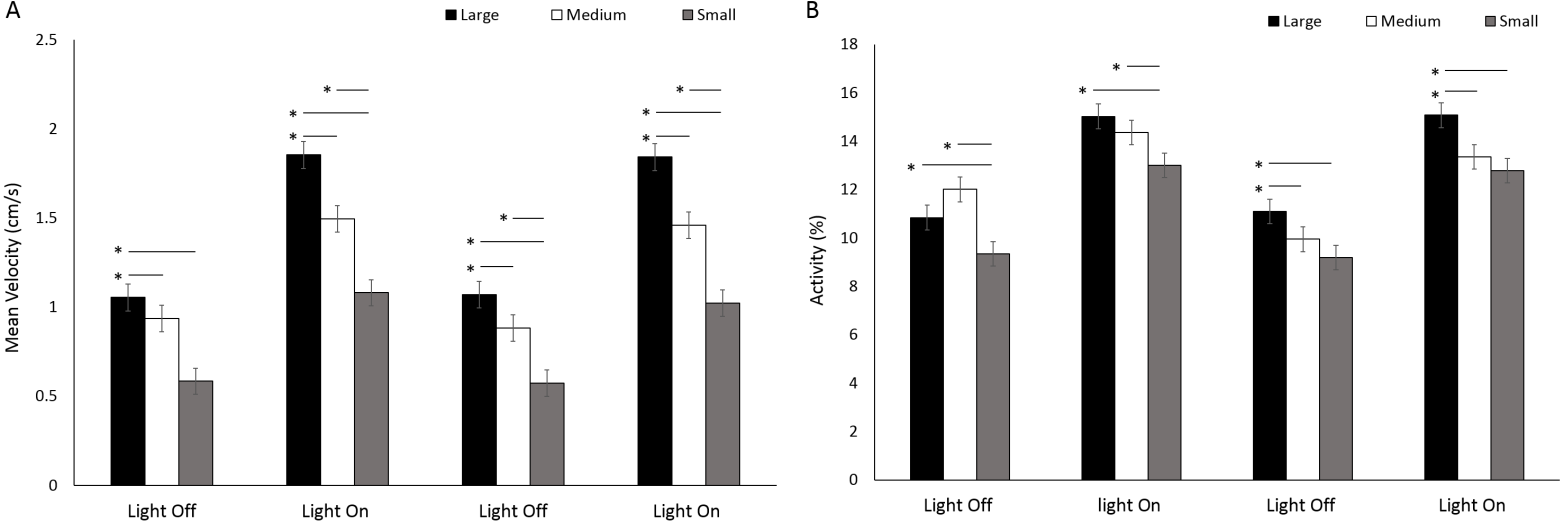
(A) Mean velocity and (B) percent activity of *G. pulex* during dark and light cycles between arena sizes. Asterisks represent significant differences between arena size (*p* < 0.05). Error bars represent 95% confidence interval.

There was a significant difference in velocity between arena sizes with a greater mean velocity reached in a large arena than medium and small (*F*(2, 414) = 109.19, *p* < 0.001; Table S3) (Fig. 10A). Mean velocity was significantly greater during light phases compared to dark (*F*(3, 1242) = 575.90, *p* < 0.001; Table S3). There was a significant interaction between size and time driven by a greater difference in velocity between arena sizes during light phases compared to dark (*F*(6, 1242) = 13.71, *p* < 0.001). Percent activity was significantly different between arena sizes as a result of greater activity levels in large arenas compared to medium or small (*F*(2, 413.8) = 21.93, *p* < 0.001; Table S3) (Fig. 10B). Activity was significantly greater during light phases compared to dark (*F*(3, 1239.1) = 431.019, *p* < 0.001; Table S3).

## Discussion

In the present study, two species of amphipod, one marine and one freshwater were observed in a range of arenas with variations in shape and/or size. Changes in three endpoints associated with locomotion were determined including spatial distribution (thigmotaxis), swimming velocity and levels of activity. It has been demonstrated that alterations in experimental design, including organism age, rearing conditions and arena size can have effects on multiple behavioural parameters including activity and thigmotaxis (Fraser et al., 2017; Cole, 1977). Furthermore, behavioural changes as a result of alterations in methodology can significantly influence the minimum effect concentrations of compounds in toxicity testing (Fraser et al., 2017). Inter-species variation in baseline behaviours is also an important factor to consider when developing behavioural assays as the observed behaviours may vary between closely related species or strains. For example, it has been demonstrated that zebrafish of different strains can show significant differences in exploration and locomotion in novel tank diving tests (Egan et al., 2009; Sackerman et al., 2010), suggesting that the strain of zebrafish used could impact the results of toxicity tests.

In this study, the marine amphipod (*E. marinus*) showed a strong preference for the outer zones, spending very little time in the central zones of both round and square arenas (4%) for the duration of trials. Some of this zonal use could be attributed to the swimming patterns of the amphipods whereby they swim until they reach an edge and continue to swim around the periphery of the zones. However, only approximately 10–15% of the overall time was spent active suggesting these amphipods display a preference for thigmotaxis (wall-hugging). Square arenas resulted in a greater proportion of time being spent in corner zones compared to walls, again suggesting the animals displayed a preference for the refuge areas created by the corners. However, this ultimately this did not have any significant effects on the zone use (inner vs outer) between arena shapes as predicted, as no difference was observed in the use of centre zones between round and square arenas. Failure of *E. marinus* to use the centre zone for the duration of the eight-minute trial makes differences in thigmotaxis between arena shapes difficult to determine in this species due to ceiling effects. The impact of floor and ceiling effects have been outlined as a limiting factor for the interpretation of some behavioural assays (Bouwknecht & Paylor, 2008). If the baseline behaviour of *E. marinus* in a laboratory assay is to always be in association with an edge, i.e., no/limited exploratory behaviour, then differences in arena shape are less likely to alter their thigmotactic response. Alternatively, if the duration of the trial was not sufficient for significant differences in thigmotactic behaviour to be observed, differences between arena shapes could not be elucidated. Current behavioural paradigms to assess exploratory behaviours, including thigmotaxis, typically rely on the novelty of the test arena, and so the behaviour of individuals is usually recorded for relatively short time periods. Recording for between 5–6 min in novel tank diving tests with fish (Stewart et al., 2011; Cachat et al., 2010; Cachat et al., 2011) and 3–5 min in open field tests with mice (Cummins & Walsh, 1976) have been found sufficient to observe significant differences in behaviour. Rosemberg et al. (2011) recorded individuals of zebrafish in a novel tank test for 15 min. Significant differences in exploratory behaviour were observed in the first 3 min of the trial and no further significant changes were observed after the first 6 min. Simon, Dupuis & Costentin (1994) tested mice in an open field for 30 min. Significant differences in thigmotaxis and locomotion were observed after the first 5 min and no further significant differences were observed after 15 min. While the trial duration in this study was in accordance with other novelty based assays , our results do not confirm whether the trial duration in this study is sufficient for *E. marinus*. Melvin et al. (2017) assessed the effects of acclimation time to a test arena on a range of swimming behaviours in mosquito fish and concluded that a longer acclimation may be required to ensure that ‘normal’ baseline behaviours are being observed. It was also demonstrated that a longer acclimation time improved the statistical power of the behavioural assay. Both of these factors can impact the environmental relevance, robustness and validity of behavioural assays, and represents an area for further work in developing the behavioural assays outlined in our present works.

We speculated that the overall activity of *E. marinus* would be greater in a round arena compared to a square, as the presence of corners would provide a refuge area which is not present in a round arena. However, despite the proportionally greater amounts of time spent in a corner zone compared to the walls of the square arena, the inverse was found with regards to activity, with a significantly greater percent activity observed in square arenas compared to round. Similar results were found in a study by Grabovskaya & Salyha (2014) whereby the effects of arena shape on multiple behavioural parameters in rats were assessed in an open field. No significant differences were reported for thigmotaxis and activity between round and square arenas (Grabovskaya & Salyha, 2014). Rats presented a trend towards a greater duration of freezing episodes in the square arenas, with episodes of freezing demonstrated in corners rather than in the centre or near walls, but this trend did not reach a level of significance.

In this study, the freshwater amphipod (*G. pulex*) was also found to exhibit thigmotactic behaviour, showing a significant preference for the periphery over central zones. Thigmotactic behaviour in *G. pulex* perhaps was not as strong as in *E. marinus*, with *G. pulex* spending 20% of the time in the centre zone compared to 3% in *E. marinus*, although they were marginally more active. Furthermore, the preferential use of corner space was not observed in *G. pulex* as with *E. marinus*. Instead, *G. pulex* spent a significantly greater amount of time against a wall compared to corners. This, however, is thought to be a result of the proportionally larger size of wall zones compared to corners, rather than a preference for walls over corners. Corner zones made up 28% of the total periphery, with walls making up 72%. Of the total duration that *G. pulex* spent in the periphery of the square arena, 34% of this was spent in a corner, and 66% was spent against a wall, making time spent in corners and walls almost directly proportional to their area. Arena shape had an effect on zone use in *G. pulex* with significantly more time spent in the centre zone of a square arena compared to round. It was expected that the presence of corners as a refuge area in square arenas would result in a reduction in the use of central zones compared to a round arena. However, the inverse was found in that the use of centre zones was greater in a square arena, with no significant preference observed for corner zones. These results suggest that the increased use of the central zone in square arenas may simply be due to its proportionally greater area than the centre zone of round arenas. Light/dark phases had an effect on zone use in *G. pulex* in a square well with less time spent in the centre zones during light phases compared to dark. The effect of light disturbance in initiating the conceivably greater thigmotactic response in *G. pulex* provides evidence that this behavioural endpoint potentially has have applications in ecotoxicological studies. The same response was found in zebrafish larvae whereby thigmotaxis was triggered by a sudden change in illumination (Schnörr et al., 2012). In the same study, thigmotaxis was significantly attenuated by an anxiolytic and enhanced by an anxiogenic drug. No significant differences were observed in the activity of *G. pulex* between round and square arenas during dark phases of the experiment and activity was greater in the square compared to round arenas during light phases, supporting the idea that *G. pulex* not pausing to use corner zones as refuge areas.

Both *E. marinus* and *G. pulex* showed a significant increase in velocity during light cycles compared to dark. Amphipods have been shown to increase velocity when exposed to a light disturbance (Bossus et al., 2014) and exhibit negative phototaxis (Guler & Ford, 2010). Exposure to anxiolytics in both of these studies report effects on velocity and phototaxis suggesting this endpoint to be a useful measure in ecotoxicology studies. Particularly as these behaviours are considered an adaptive response to avoid predation (Bauer et al., 2005; Bethel & Holmes, 1973; Bakker, Mazzi & Zala, 1997) providing a link to ecological effects of behaviour. No differences were observed between square and round arenas for velocity during light phases for both amphipod species. No differences in velocity were observed between arena shapes during dark phases for *G. pulex*. Comparatively, *E. marinus* exhibited a greater mean velocity in square arenas compared to round during dark phases. This may be a result of size differences between *G. pulex* and *E. marinus*. Arena size was found to have an effect on velocity in *G. pulex* whereby increasing area resulted in increased velocity, however, these organisms would eventually reach a point of maximum velocity whereby further increasing arena size would have no effect on swimming speed. The square arena had a greater area than the round which may have allowed *E. marinus* to reach a greater mean velocity.

The overall ratios of the test arenas areas (mm2) were 1: 2.4: 6 (small: medium: large) with equal proportions of the inner (33.3%) vs outer (66.7%) zones. Arena size had significant effects on zone use in *G. pulex* in the larger arenas using the inner zones for a greater proportion of the overall time compared to both the small and medium sized arenas which did not differ. The opposite has been found in voles in an open field test (Eilam, 2003). Voles were tested for spatial distribution and activity under a range of arena sizes. It was found that these animals showed a preference for peripheral areas and greater avoidance of the centre zone with increasing arena size. Foraging behaviours in cockroaches have also been found to be affected by arena size with the use of refuge areas increasing with increased arena size (Le Patourel, 1998). The opposing results in our study compared to that reported in the literature would suggest that the increased use of outer areas in smaller arenas is more likely due to an increased probability of hitting an edge in a smaller arena whilst swimming rather that a preference for edges in smaller arenas. Mean velocity and the percentage time spent active in *G. pulex* were also dependent on the size of round arenas, with greater arenas evoking greater swimming speeds and time spent active. This has also been observed in *Drosophila melanogaster* whose initial activity levels increased linearly with the circumference of a circular arena (Liu, Davis & Roman, 2007) and was suggested as an increase in exploratory behaviour with increasing space to explore. The same has been observed in rats (Montgomery, 1951) and gerbils (Oldham & Morlock, 1970) whereby activity increased in an open field test with increasing arena size.

## Conclusions

This study has provided evidence that amphipods exhibit a range of measurable behaviours potentially associated with anxiety. Differences in zone use and spatial distribution between the two amphipod species suggest that care should be taken when selecting your test species for use in behavioural ecotoxicological studies as this could potentially affect the interpretation of toxicity tests. The greater use of outer zones in *E. marinus* has the potential for producing a ‘ceiling effect’ thereby making the ability to measure ‘exploratory’ behaviours in this test arena less feasible. *G. pulex*, on the other hand, represents a potential invertebrate candidate for assessing thigmotaxis due to its lower overall baseline thigmotactic behaviour which can be enhanced by exposure to the light stimulus. Arena shape can have an effect on zone use, velocity and activity in amphipods with different shaped wells altering the use the outer zones which corner refuges are present. Arena size was found to have a direct effect on all behaviours assessed in *G. pulex* with greater arena size resulting in more time spent in the central zones, greater mean velocity and overall activity. This has implications when trying to develop assays for high throughput assessment whereby behaviours may be lost if the organism is not given ‘space to behave’. In this instance, researchers may interpret negative findings as insignificant effects of chemical exposure when in fact an inappropriate arena may be preventing behaviours. This study has highlighted multiple behaviours with amphipods that show potential for use in behavioural ecotoxicology. Both amphipods engaged in thigmotaxis via a strong association with outer zones during behavioural trials, further work would require validation with anxiolytic and anxiogenic compounds to determine if the thigmotactic behaviour in this species is associated with anxiety behaviours or a result of edge effects. Further work would be required for both species to determine the size at which further increasing the area has no effect on the behaviours measured. It is also suggested that future work includes the optimisation of other factors that may impact anxiety-like behaviours in novelty tests including pre-test animal housing (Parker et al., 2012; Blaser & Rosemberg, 2012), effects of habituation (Maximino et al., 2010a), length of acclimation, and duration of light/dark phases.

##  Supplemental Information

10.7717/peerj.5271/supp-1 Supplemental Information 1 Tables S1-S3

## Additional Information and Declarations

### Data Availability

The following information was supplied regarding data availability: Ford A, Kohler S, Parker MO. 2018. “Shape and Size of the Arenas Affect Amphipod Behaviours.” Open Science Framework. July 3. https://osf.io/j7b52/.
