# Supplementary material for: Shape and size of the arenas affect amphipod behaviours: implications for ecotoxicology
Source: PeerJ. 2018 Jul 26;6:e5271. doi: 10.7717/peerj.5271 (PMC6064634; doi:10.7717/peerj.5271)
Supplement: Supplemental Information 1 [file peerj-06-5271-s001.docx]

| S-Table 1: Bonferroni corrections representing main effects and interactions between zones and time bins in a square arena for *E. marinus* and *G. pulex.* Interaction numbers represent time bins. i.e. 2-4 represents a comparison between minute 2 and minute 4 of the experiment. | | | | | | | |
| --- | --- | --- | --- | --- | --- | --- | --- |
| Species | **Comparison** | **Interaction** | **df** | **std. error** | **P-value** | **lower bound** | **upper bound** |
| *E. marinus* | **Centre** | 2-4 | -0.14 | 0.85 | 1.000 | -2.39 | 2.12 |
|  |  | 2-6 | -0.24 | 0.85 | 1.000 | -2.50 | 2.01 |
|  |  | 2-8 | -0.52 | 0.85 | 1.000 | -2.78 | 1.73 |
|  |  | 4-6 | -0.10 | 0.85 | 1.000 | -2.36 | 2.15 |
|  |  | 4-8 | -0.39 | 0.85 | 1.000 | -2.64 | 1.87 |
|  |  | 6-8 | -0.28 | 0.85 | 1.000 | -2.54 | 1.97 |
|  | **Corner** | 2-4 | -5.68 | 2.16 | 0.052 | -11.39 | 0.03 |
|  |  | 2-6 | -1.90 | 2.16 | 1.000 | -7.61 | 3.81 |
|  |  | 2-8 | -6.96 | 2.16 | 0.008 | -12.68 | -1.25 |
|  |  | 4-6 | 3.78 | 2.16 | 0.481 | -1.93 | 9.49 |
|  |  | 4-8 | -1.29 | 2.16 | 1.000 | -7.00 | 4.42 |
|  |  | 6-8 | -5.07 | 2.16 | 0.115 | -10.78 | 0.64 |
|  | **Wall** | 2-4 | 5.63 | 1.81 | 0.012 | 0.84 | 10.41 |
|  |  | 2-6 | 1.95 | 1.81 | 1.000 | -2.83 | 6.73 |
|  |  | 2-8 | 7.30 | 1.81 | <0.001 | 2.52 | 12.08 |
|  |  | 4-6 | -3.68 | 1.81 | 0.253 | -8.46 | 1.10 |
|  |  | 4-8 | 1.67 | 1.81 | 1.000 | -3.11 | 6.45 |
|  |  | 6-8 | 5.35 | 1.81 | 0.019 | 0.57 | 10.13 |
|  | **2min** | centre - corner | -47.32 | 1.38 | <0.001 | -50.65 | -44.00 |
|  |  | centre - wall | -41.00 | 1.38 | <0.001 | -44.32 | -37.68 |
|  |  | corner - wall | 6.32 | 1.40 | <0.001 | 2.96 | 9.68 |
|  | **4 min** | centre - corner | -53.20 | 1.63 | <0.001 | -57.11 | -49.28 |
|  |  | centre - wall | -35.28 | 1.63 | <0.001 | -39.20 | -31.37 |
|  |  | corner - wall | 17.91 | 1.63 | <0.001 | 14.00 | 21.83 |
|  | **6 min** | centre - corner | -48.91 | 1.53 | <0.001 | -52.58 | -45.24 |
|  |  | centre - wall | -38.38 | 1.53 | <0.001 | -42.05 | -34.71 |
|  |  | corner - wall | 10.53 | 1.53 | <0.001 | 6.86 | 14.21 |
|  | **8 min** | centre - corner | -53.30 | 1.74 | <0.001 | -57.49 | -49.11 |
|  |  | centre - wall | -33.62 | 1.73 | <0.001 | -37.79 | -29.45 |
|  |  | corner - wall | 19.69 | 1.74 | <0.001 | 15.51 | 23.87 |
| *G. pulex* | **Centre** | 2-4 | 6.34 | 2.12 | 0.017 | 0.73 | 11.96 |
|  |  | 2-6 | -6.32 | 2.12 | 0.018 | -11.94 | -0.71 |
|  |  | 2-8 | 2.99 | 2.12 | 0.955 | -2.63 | 8.60 |
|  |  | 4-6 | -12.67 | 2.12 | <0.001 | -18.28 | -7.05 |
|  |  | 4-8 | -3.36 | 2.12 | 0.683 | -8.97 | 2.26 |
|  |  | 6-8 | 9.31 | 2.12 | <0.001 | 3.70 | 14.92 |
|  | **Corner** | 2-4 | -8.27 | 1.74 | <0.001 | -12.88 | -3.65 |
|  |  | 2-6 | 1.21 | 1.74 | 1.000 | -3.40 | 5.82 |
|  |  | 2-8 | -5.78 | 1.74 | 0.006 | -10.39 | -1.16 |
|  |  | 4-6 | 9.48 | 1.74 | <0.001 | 4.87 | 14.09 |
|  |  | 4-8 | 2.49 | 1.74 | 0.918 | -2.12 | 7.10 |
|  |  | 6-8 | -6.99 | 1.74 | <0.001 | -11.60 | -2.38 |
|  | **Wall** | 2-4 | 1.92 | 1.72 | 1.000 | -2.64 | 6.49 |
|  |  | 2-6 | 5.11 | 1.72 | 0.019 | 0.55 | 9.67 |
|  |  | 2-8 | 2.79 | 1.72 | 0.638 | -1.78 | 7.35 |
|  |  | 4-6 | 3.19 | 1.72 | 0.389 | -1.38 | 7.75 |
|  |  | 4-8 | 0.87 | 1.72 | 1.000 | -3.70 | 5.43 |
|  |  | 6-8 | -2.32 | 1.72 | 1.000 | -6.89 | 2.24 |
|  | **2 min** | centre - corner | -4.66 | 2.05 | 0.071 | -9.59 | 0.27 |
|  |  | centre - wall | -35.41 | 2.04 | <0.001 | -40.33 | -30.49 |
|  |  | corner - wall | -30.75 | 2.04 | <0.001 | -35.65 | -25.85 |
|  | **4 min** | centre - corner | -18.18 | 1.41 | <0.001 | -21.56 | -14.80 |
|  |  | centre - wall | -38.25 | 1.41 | <0.001 | -41.63 | -34.87 |
|  |  | corner - wall | -20.07 | 1.41 | <0.001 | -23.45 | -16.68 |
|  | **6 min** | centre - corner | 3.02 | 2.08 | 0.443 | -1.98 | 8.02 |
|  |  | centre - wall | -23.74 | 2.07 | <0.001 | -28.74 | -18.75 |
|  |  | corner - wall | -26.76 | 2.07 | <0.001 | -31.73 | -21.79 |
|  | **8 min** | centre - corner | -13.43 | 1.46 | <0.001 | -16.94 | -9.92 |
|  |  | centre - wall | -35.12 | 1.46 | <0.001 | -38.63 | -31.61 |
|  |  | corner - wall | -21.69 | 1.45 | <0.001 | -25.19 | -18.19 |

| S-Table 2: Bonferroni corrections representing main effects and interactions between arena shapes and time bins on thigmotaxis, mean velocity and percent activity for *E. marinus* and *G. pulex.* interaction numbers represent time bins. i.e. 2-4 represents a comparison between minute 2 and minute 4 of the experiment. | | | | | | | | | | | | | | | | |
| --- | --- | --- | --- | --- | --- | --- | --- | --- | --- | --- | --- | --- | --- | --- | --- | --- |
| Species | **Endpoint** | | **Comparison** | | **Interaction** | | | **df** | | **std. error** | | **sig** | **lower bound** | | **upper bound** | |
| *E. marinus* | **Thigmotaxis** | | **Round** | | 2-4 | | | 0.76 | | 1.01 | | 1.000 | -1.90 | | 3.43 | |
|  |  | |  | | 2-6 | | | -0.26 | | 1.01 | | 1.000 | -2.93 | | 2.41 | |
|  |  | |  | | 2-8 | | | -0.44 | | 1.01 | | 1.000 | -3.10 | | 2.23 | |
|  |  | |  | | 4-6 | | | -1.02 | | 1.01 | | 1.000 | -3.69 | | 1.64 | |
|  |  | |  | | 4-8 | | | -1.20 | | 1.01 | | 1.000 | -3.87 | | 1.47 | |
|  |  | |  | | 6-8 | | | -0.18 | | 1.01 | | 1.000 | -2.84 | | 2.49 | |
|  |  | | **Square** | | 2-4 | | | -0.14 | | 0.85 | | 1.000 | -2.39 | | 2.12 | |
|  |  | |  | | 2-6 | | | -0.24 | | 0.85 | | 1.000 | -2.50 | | 2.01 | |
|  |  | |  | | 2-8 | | | -0.52 | | 0.85 | | 1.000 | -2.78 | | 1.73 | |
|  |  | |  | | 4-6 | | | -0.10 | | 0.85 | | 1.000 | -2.36 | | 2.15 | |
|  |  | |  | | 4-8 | | | -0.39 | | 0.85 | | 1.000 | -2.64 | | 1.87 | |
|  |  | |  | | 6-8 | | | -0.28 | | 0.85 | | 1.000 | -2.54 | | 1.97 | |
|  |  | | **2min** | | round-square | | | 0.54 | | 0.82 | | 0.508 | -1.07 | | 2.16 | |
|  |  | | **4min** | | round-square | | | -0.36 | | 0.84 | | 0.670 | -2.02 | | 1.30 | |
|  |  | | **6min** | | round-square | | | 0.56 | | 0.96 | | 0.562 | -1.34 | | 2.46 | |
|  |  | | **8min** | | round-square | | | 0.45 | | 1.08 | | 0.674 | -1.67 | | 2.58 | |
|  | **Velocity** | | **Round** | | 2-4 | | | -0.89 | | 0.09 | | <0.001 | -1.14 | | -0.65 | |
|  |  | |  | | 2-6 | | | 0.06 | | 0.09 | | 1.000 | -0.18 | | 0.30 | |
|  |  | |  | | 2-8 | | | -0.38 | | 0.09 | | <0.001 | -0.62 | | -0.14 | |
|  |  | |  | | 4-6 | | | 0.95 | | 0.09 | | <0.001 | 0.71 | | 1.20 | |
|  |  | |  | | 4-8 | | | 0.52 | | 0.09 | | <0.001 | 0.27 | | 0.76 | |
|  |  | |  | | 6-8 | | | -0.44 | | 0.09 | | <0.001 | -0.68 | | -0.20 | |
|  |  | | **Square** | | 2-4 | | | -0.44 | | 0.07 | | <0.001 | -0.62 | | -0.25 | |
|  |  | |  | | 2-6 | | | -0.10 | | 0.07 | | 0.889 | -0.29 | | 0.08 | |
|  |  | |  | | 2-8 | | | -0.19 | | 0.07 | | 0.037 | -0.38 | | -0.01 | |
|  |  | |  | | 4-6 | | | 0.34 | | 0.07 | | <0.001 | 0.15 | | 0.52 | |
|  |  | |  | | 4-8 | | | 0.25 | | 0.07 | | 0.003 | 0.06 | | 0.43 | |
|  |  | |  | | 6-8 | | | -0.09 | | 0.07 | | 1.000 | -0.28 | | 0.09 | |
|  |  | | **2min** | | round-square | | | -0.26 | | 0.07 | | <0.001 | -0.39 | | -0.12 | |
|  |  | | **4min** | | round-square | | | 0.20 | | 0.09 | | 0.033 | 0.02 | | 0.38 | |
|  |  | | **6min** | | round-square | | | -0.42 | | 0.07 | | <0.001 | -0.57 | | -0.27 | |
|  |  | | **8min** | | round-square | | | -0.07 | | 0.09 | | 0.416 | -0.24 | | 0.10 | |
|  | **Activity** | | **Round** | | 2-4 | | | -2.01 | | 0.37 | | <0.001 | -3.00 | | -1.02 | |
|  |  | |  | | 2-6 | | | 0.38 | | 0.37 | | 1.000 | -0.61 | | 1.36 | |
|  |  | |  | | 2-8 | | | -0.16 | | 0.37 | | 1.000 | -1.15 | | 0.83 | |
|  |  | |  | | 4-6 | | | 2.38 | | 0.37 | | <0.001 | 1.39 | | 3.37 | |
|  |  | |  | | 4-8 | | | 1.85 | | 0.37 | | <0.001 | 0.86 | | 2.84 | |
|  |  | |  | | 6-8 | | | -0.53 | | 0.37 | | 0.931 | -1.52 | | 0.46 | |
|  |  | | **Square** | | 2-4 | | | -1.59 | | 0.40 | | <0.001 | -2.64 | | -0.55 | |
|  |  | |  | | 2-6 | | | -0.45 | | 0.40 | | 1.000 | -1.49 | | 0.60 | |
|  |  | |  | | 2-8 | | | -0.48 | | 0.40 | | 1.000 | -1.53 | | 0.57 | |
|  |  | |  | | 4-6 | | | 1.15 | | 0.40 | | 0.023 | 0.10 | | 2.20 | |
|  |  | |  | | 4-8 | | | 1.11 | | 0.40 | | 0.031 | 0.06 | | 2.16 | |
|  |  | |  | | 6-8 | | | -0.04 | | 0.40 | | 1.000 | -1.08 | | 1.01 | |
|  |  | | **2min** | | round-square | | | -1.79 | | 0.37 | | <0.001 | -2.52 | | -1.06 | |
|  |  | | **4min** | | round-square | | | -1.38 | | 0.38 | | <0.001 | -2.12 | | -0.64 | |
|  |  | | **6min** | | round-square | | | -2.61 | | 0.41 | | <0.001 | -3.41 | | -1.80 | |
|  |  | | **8min** | | round-square | | | -2.11 | | 0.38 | | <0.001 | -2.87 | | -1.36 | |
| *G. pulex* | **Thigmotaxis** | | **Round** | | 2-4 | | | 1.31 | | 1.42 | | 1.000 | -2.44 | | 5.05 | |
|  |  | |  | | 2-6 | | | -5.29 | | 1.42 | | 0.001 | -9.03 | | -1.54 | |
|  |  | |  | | 2-8 | | | 0.53 | | 1.42 | | 1.000 | -3.22 | | 4.27 | |
|  |  | |  | | 4-6 | | | -6.59 | | 1.42 | | <0.001 | -10.34 | | -2.84 | |
|  |  | |  | | 4-8 | | | -0.78 | | 1.42 | | 1.000 | -4.53 | | 2.97 | |
|  |  | |  | | 6-8 | | | 5.81 | | 1.42 | | <0.001 | 2.06 | | 9.56 | |
|  |  | | **Square** | | 2-4 | | | 6.34 | | 2.12 | | 0.017 | 0.73 | | 11.96 | |
|  |  | |  | | 2-6 | | | -6.32 | | 2.12 | | 0.018 | -11.94 | | -0.71 | |
|  |  | |  | | 2-8 | | | 2.99 | | 2.12 | | 0.955 | -2.63 | | 8.60 | |
|  |  | |  | | 4-6 | | | -12.67 | | 2.12 | | <0.001 | -18.28 | | -7.05 | |
|  |  | |  | | 4-8 | | | -3.36 | | 2.12 | | 0.683 | -8.97 | | 2.26 | |
|  |  | |  | | 6-8 | | | 9.31 | | 2.12 | | <0.001 | 3.70 | | 14.92 | |
|  |  | | **2min** | | round-square | | | -10.24 | | 2.05 | | <0.001 | -14.27 | | -6.20 | |
|  |  | | **4min** | | round-square | | | -5.20 | | 1.08 | | <0.001 | -7.33 | | -3.06 | |
|  |  | | **6min** | | round-square | | | -11.27 | | 2.27 | | <0.001 | -15.75 | | -6.80 | |
|  |  | | **8min** | | round-square | | | -7.77 | | 1.35 | | <0.001 | -10.44 | | -5.11 | |
|  | **Velocity** | | **Round** | | 2-4 | | | -0.80 | | 0.07 | | <0.001 | -0.97 | | -0.63 | |
|  |  | |  | | 2-6 | | | -0.02 | | 0.07 | | 1.000 | -0.19 | | 0.16 | |
|  |  | |  | | 2-8 | | | -0.79 | | 0.07 | | <0.001 | -0.96 | | -0.62 | |
|  |  | |  | | 4-6 | | | 0.78 | | 0.07 | | <0.001 | 0.61 | | 0.96 | |
|  |  | |  | | 4-8 | | | 0.01 | | 0.07 | | 1.000 | -0.16 | | 0.18 | |
|  |  | |  | | 6-8 | | | -0.77 | | 0.07 | | <0.001 | -0.94 | | -0.60 | |
|  |  | | **Square** | | 2-4 | | | -0.89 | | 0.09 | | <0.001 | -1.12 | | -0.66 | |
|  |  | |  | | 2-6 | | | -0.12 | | 0.09 | | 1.000 | -0.35 | | 0.11 | |
|  |  | |  | | 2-8 | | | -0.85 | | 0.09 | | <0.001 | -1.09 | | -0.62 | |
|  |  | |  | | 4-6 | | | 0.77 | | 0.09 | | <0.001 | 0.53 | | 1.00 | |
|  |  | |  | | 4-8 | | | 0.04 | | 0.09 | | 1.000 | -0.20 | | 0.27 | |
|  |  | |  | | 6-8 | | | -0.73 | | 0.09 | | <0.001 | -0.96 | | -0.50 | |
|  |  | | **2min** | | round-square | | | 0.16 | | 0.07 | | 0.027 | 0.02 | | 0.31 | |
|  |  | | **4min** | | round-square | | | 0.07 | | 0.08 | | 0.361 | -0.09 | | 0.23 | |
|  |  | | **6min** | | round-square | | | 0.06 | | 0.07 | | 0.425 | -0.08 | | 0.20 | |
|  |  | | **8min** | | round-square | | | 0.10 | | 0.08 | | 0.216 | -0.06 | | 0.25 | |
|  | **Activity** | | **Round** | | 2-4 | | | -4.18 | | 0.36 | | <0.001 | -5.13 | | -3.24 | |
|  |  | |  | | 2-6 | | | -0.25 | | 0.36 | | 1.000 | -1.20 | | 0.69 | |
|  |  | |  | | 2-8 | | | -4.23 | | 0.36 | | <0.001 | -5.18 | | -3.28 | |
|  |  | |  | | 4-6 | | | 3.93 | | 0.36 | | <0.001 | 2.98 | | 4.88 | |
|  |  | |  | | 4-8 | | | -0.05 | | 0.36 | | 1.000 | -0.99 | | 0.90 | |
|  |  | |  | | 6-8 | | | -3.98 | | 0.36 | | <0.001 | -4.92 | | -3.03 | |
|  |  | | **Square** | | 2-4 | | | -7.02 | | 0.84 | | <0.001 | -9.24 | | -4.80 | |
|  |  | |  | | 2-6 | | | -1.30 | | 0.84 | | 0.730 | -3.53 | | 0.92 | |
|  |  | |  | | 2-8 | | | -7.13 | | 0.84 | | <0.001 | -9.35 | | -4.91 | |
|  |  | |  | | 4-6 | | | 5.72 | | 0.84 | | <0.001 | 3.50 | | 7.94 | |
|  |  | |  | | 4-8 | | | -0.11 | | 0.84 | | 1.000 | -2.33 | | 2.12 | |
|  |  | |  | | 6-8 | | | -5.83 | | 0.84 | | <0.001 | -8.05 | | -3.60 | |
|  |  | | **2min** | | round-square | | | -0.51 | | 0.59 | | 0.381 | -1.67 | | 0.64 | |
|  |  | | **4min** | | round-square | | | -3.35 | | 0.63 | | <0.001 | -4.60 | | -2.11 | |
|  |  | | **6min** | | round-square | | | -1.56 | | 0.57 | | 0.006 | -2.67 | | -0.45 | |
|  |  | | **8min** | | round-square | | | -3.41 | | 0.67 | | <0.001 | -4.72 | | -2.10 | |
| S-Table 3: Bonferroni corrections representing main effects and interactions between arena size and time bins on thigmotaxis, mean velocity and percent activity in large, medium and small arenas *G. pulex.* Interaction numbers represent time bins. i.e. 2-4 represents a comparison between minute 2 and minute 4 of the experiment, letters represent small, medium and large arenas. | | | | | | | | | | | | | | | |  |
| Endpoint | | **Comparison** | | **Interaction** | | **df** | **std. error** | | **P-value** | | **lower bound** | | | **upper bound** | |  |
| Thigmotaxis | | **Large** | | 2-4 | | 1.31 | 1.42 | | 1.000 | | -2.44 | | | 5.05 | |  |
|  | |  | | 2-6 | | -5.29 | 1.42 | | 0.001 | | -9.03 | | | -1.54 | |  |
|  | |  | | 2-8 | | 0.53 | 1.42 | | 1.000 | | -3.22 | | | 4.27 | |  |
|  | |  | | 4-6 | | -6.59 | 1.42 | | <0.001 | | -10.34 | | | -2.84 | |  |
|  | |  | | 4-8 | | -0.78 | 1.42 | | 1.000 | | -4.53 | | | 2.97 | |  |
|  | |  | | 6-8 | | 5.81 | 1.42 | | <0.001 | | 2.06 | | | 9.56 | |  |
|  | | **Medium** | | 2-4 | | 2.87 | 0.69 | | <0.001 | | 1.04 | | | 4.70 | |  |
|  | |  | | 2-6 | | -1.10 | 0.69 | | 0.682 | | -2.93 | | | 0.74 | |  |
|  | |  | | 2-8 | | 2.31 | 0.69 | | 0.005 | | 0.48 | | | 4.14 | |  |
|  | |  | | 4-6 | | -3.97 | 0.69 | | <0.001 | | -5.80 | | | -2.14 | |  |
|  | |  | | 4-8 | | -0.56 | 0.69 | | 1.000 | | -2.40 | | | 1.27 | |  |
|  | |  | | 6-8 | | 3.40 | 0.69 | | <0.001 | | 1.57 | | | 5.23 | |  |
|  | | **Small** | | 2-4 | | -0.81 | 1.22 | | 1.000 | | -4.03 | | | 2.42 | |  |
|  | |  | | 2-6 | | -1.37 | 1.22 | | 1.000 | | -4.59 | | | 1.86 | |  |
|  | |  | | 2-8 | | -0.15 | 1.22 | | 1.000 | | -3.37 | | | 3.08 | |  |
|  | |  | | 4-6 | | -0.56 | 1.22 | | 1.000 | | -3.79 | | | 2.66 | |  |
|  | |  | | 4-8 | | 0.66 | 1.22 | | 1.000 | | -2.57 | | | 3.88 | |  |
|  | |  | | 6-8 | | 1.22 | 1.22 | | 1.000 | | -2.01 | | | 4.44 | |  |
|  | | **2 min** | | L-M | | 3.68 | 0.81 | | <0.001 | | 1.74 | | | 5.63 | |  |
|  | |  | | L-S | | 5.35 | 0.81 | | <0.001 | | 3.39 | | | 7.30 | |  |
|  | |  | | M-S | | 1.66 | 0.80 | | 0.115 | | -0.26 | | | 3.59 | |  |
|  | | **4 min** | | L-M | | 6.87 | 0.67 | | <0.001 | | 5.25 | | | 8.49 | |  |
|  | |  | | L-S | | 4.14 | 0.67 | | <0.001 | | 2.51 | | | 5.76 | |  |
|  | |  | | M-S | | -2.74 | 0.67 | | <0.001 | | -4.35 | | | -1.13 | |  |
|  | | **6 min** | | L-M | | 7.40 | 0.99 | | <0.001 | | 5.03 | | | 9.77 | |  |
|  | |  | | L-S | | 7.93 | 0.99 | | <0.001 | | 5.56 | | | 10.30 | |  |
|  | |  | | M-S | | 0.53 | 0.97 | | 1.000 | | -1.80 | | | 2.86 | |  |
|  | | **8 min** | | L-M | | 6.94 | 0.68 | | <0.001 | | 5.29 | | | 8.59 | |  |
|  | |  | | L-S | | 5.53 | 0.69 | | <0.001 | | 3.88 | | | 7.18 | |  |
|  | |  | | M-S | | -1.41 | 0.68 | | 0.119 | | -3.05 | | | 0.23 | |  |
| Velocity | | **Large** | | 2-4 | | -0.80 | 0.07 | | <0.001 | | -0.97 | | | -0.63 | |  |
|  | |  | | 2-6 | | -0.02 | 0.07 | | 1.000 | | -0.19 | | | 0.16 | |  |
|  | |  | | 2-8 | | -0.79 | 0.07 | | <0.001 | | -0.96 | | | -0.62 | |  |
|  | |  | | 4-6 | | 0.78 | 0.07 | | <0.001 | | 0.61 | | | 0.96 | |  |
|  | |  | | 4-8 | | 0.01 | 0.07 | | 1.000 | | -0.16 | | | 0.18 | |  |
|  | |  | | 6-8 | | -0.77 | 0.07 | | <0.001 | | -0.94 | | | -0.60 | |  |
|  | | **Medium** | | 2-4 | | -0.56 | 0.05 | | <0.001 | | -0.69 | | | -0.42 | |  |
|  | |  | | 2-6 | | 0.06 | 0.05 | | 1.000 | | -0.08 | | | 0.19 | |  |
|  | |  | | 2-8 | | -0.52 | 0.05 | | <0.001 | | -0.66 | | | -0.38 | |  |
|  | |  | | 4-6 | | 0.61 | 0.05 | | <0.001 | | 0.48 | | | 0.75 | |  |
|  | |  | | 4-8 | | 0.04 | 0.05 | | 1.000 | | -0.10 | | | 0.17 | |  |
|  | |  | | 6-8 | | -0.58 | 0.05 | | <0.001 | | -0.71 | | | -0.44 | |  |
|  | | **Small** | | 2-4 | | -0.50 | 0.04 | | <0.001 | | -0.61 | | | -0.38 | |  |
|  | |  | | 2-6 | | 0.01 | 0.04 | | 1.000 | | -0.10 | | | 0.13 | |  |
|  | |  | | 2-8 | | -0.44 | 0.04 | | <0.001 | | -0.55 | | | -0.32 | |  |
|  | |  | | 4-6 | | 0.51 | 0.04 | | <0.001 | | 0.39 | | | 0.62 | |  |
|  | |  | | 4-8 | | 0.06 | 0.04 | | 1.000 | | -0.06 | | | 0.17 | |  |
|  | |  | | 6-8 | | -0.45 | 0.04 | | <0.001 | | -0.56 | | | -0.33 | |  |
|  | | **2 min** | | L-M | | 0.12 | 0.06 | | 0.112 | | -0.02 | | | 0.26 | |  |
|  | |  | | L-S | | 0.47 | 0.06 | | <0.001 | | 0.33 | | | 0.61 | |  |
|  | |  | | M-S | | 0.35 | 0.06 | | <0.001 | | 0.22 | | | 0.49 | |  |
|  | | **4 min** | | L-M | | 0.36 | 0.05 | | <0.001 | | 0.23 | | | 0.49 | |  |
|  | |  | | L-S | | 0.77 | 0.05 | | <0.001 | | 0.64 | | | 0.90 | |  |
|  | |  | | M-S | | 0.41 | 0.05 | | <0.001 | | 0.28 | | | 0.54 | |  |
|  | | **6 min** | | L-M | | 0.19 | 0.05 | | 0.001 | | 0.06 | | | 0.32 | |  |
|  | |  | | L-S | | 0.50 | 0.05 | | <0.001 | | 0.37 | | | 0.62 | |  |
|  | |  | | M-S | | 0.31 | 0.05 | | <0.001 | | 0.18 | | | 0.43 | |  |
|  | | **8 min** | | L-M | | 0.39 | 0.05 | | <0.001 | | 0.26 | | | 0.51 | |  |
|  | |  | | L-S | | 0.82 | 0.05 | | <0.001 | | 0.69 | | | 0.95 | |  |
|  | |  | | M-S | | 0.43 | 0.05 | | <0.001 | | 0.31 | | | 0.56 | |  |
| Activity | | **Large** | | 2-4 | | -4.18 | 0.36 | | <0.001 | | -5.13 | | | -3.24 | |  |
|  | |  | | 2-6 | | -0.25 | 0.36 | | 1.000 | | -1.20 | | | 0.69 | |  |
|  | |  | | 2-8 | | -4.23 | 0.36 | | <0.001 | | -5.18 | | | -3.28 | |  |
|  | |  | | 4-6 | | 3.93 | 0.36 | | <0.001 | | 2.98 | | | 4.88 | |  |
|  | |  | | 4-8 | | -0.05 | 0.36 | | 1.000 | | -0.99 | | | 0.90 | |  |
|  | |  | | 6-8 | | -3.98 | 0.36 | | <0.001 | | -4.92 | | | -3.03 | |  |
|  | | **Medium** | | 2-4 | | -2.42 | 0.45 | | <0.001 | | -3.62 | | | -1.22 | |  |
|  | |  | | 2-6 | | 2.14 | 0.45 | | <0.001 | | 0.94 | | | 3.34 | |  |
|  | |  | | 2-8 | | -1.25 | 0.45 | | 0.035 | | -2.45 | | | -0.05 | |  |
|  | |  | | 4-6 | | 4.56 | 0.45 | | <0.001 | | 3.36 | | | 5.76 | |  |
|  | |  | | 4-8 | | 1.17 | 0.45 | | 0.060 | | -0.03 | | | 2.37 | |  |
|  | |  | | 6-8 | | -3.39 | 0.45 | | <0.001 | | -4.59 | | | -2.19 | |  |
|  | | **Small** | | 2-4 | | -3.65 | 0.30 | | <0.001 | | -4.44 | | | -2.87 | |  |
|  | |  | | 2-6 | | 0.15 | 0.30 | | 1.000 | | -0.64 | | | 0.93 | |  |
|  | |  | | 2-8 | | -3.44 | 0.30 | | <0.001 | | -4.22 | | | -2.65 | |  |
|  | |  | | 4-6 | | 3.80 | 0.30 | | <0.001 | | 3.01 | | | 4.58 | |  |
|  | |  | | 4-8 | | 0.22 | 0.30 | | 1.000 | | -0.57 | | | 1.00 | |  |
|  | |  | | 6-8 | | -3.58 | 0.30 | | <0.001 | | -4.37 | | | -2.79 | |  |
|  | | **2 min** | | L-M | | -1.24 | 0.40 | | 0.006 | | -2.20 | | | -0.28 | |  |
|  | |  | | L-S | | 1.50 | 0.40 | | 0.001 | | 0.54 | | | 2.46 | |  |
|  | |  | | M-S | | 2.74 | 0.40 | | <0.001 | | 1.78 | | | 3.69 | |  |
|  | | **4 min** | | L-M | | 0.52 | 0.40 | | 0.596 | | -0.45 | | | 1.49 | |  |
|  | |  | | L-S | | 2.03 | 0.40 | | <0.001 | | 1.06 | | | 3.00 | |  |
|  | |  | | M-S | | 1.51 | 0.40 | | 0.001 | | 0.54 | | | 2.47 | |  |
|  | | **6 min** | | L-M | | 1.15 | 0.33 | | 0.002 | | 0.36 | | | 1.95 | |  |
|  | |  | | L-S | | 1.90 | 0.33 | | <0.001 | | 1.10 | | | 2.69 | |  |
|  | |  | | M-S | | 0.75 | 0.33 | | 0.072 | | -0.04 | | | 1.54 | |  |
|  | | **8 min** | | L-M | | 1.74 | 0.36 | | <0.001 | | 0.87 | | | 2.61 | |  |
|  | |  | | L-S | | 2.29 | 0.36 | | <0.001 | | 1.43 | | | 3.16 | |  |
|  | |  | | M-S | | 0.55 | 0.36 | | 0.371 | | -0.31 | | | 1.42 | |  |
